# Supplementary material for: Adequate immune response ensured by binary IL-2 and graded CD25 expression in a murine transfer model
Source: eLife. 2016 Dec 30;5:e20616. doi: 10.7554/eLife.20616 (PMC5201416; doi:10.7554/eLife.20616)
Supplement: Supplementary file 1: — Parameter names, symbols and values used for the mathematical modeling are given. DOI: http://dx.doi.org/10.7554/eLife.20616.015 [file elife-20616-supp1.doc]

Supplementary File 1: Parameter estimates for the mathematical model.

Parameter names, symbols and values used for the mathematical modeling are given.

| **Parameter** | **Symbol** | **Value** | **Reference** |
| --- | --- | --- | --- |
| cell radius | *rCell* | 5 µm | Chapman et al. 1981 |
| mean cell-to-cell distance | *dCell* | 15 µm | Lischke et al. 2012 |
| IL-2 consumption by unspecific Tregs | *dUR* | 0.56 min^−1^ | own estimation |
| size of T-Cell area | *l* | 300 µm | Mueller and Germain 2009 |
| IL-2 diffusion coefficient | *D* | 420 µM^2^min^−1^ | Lower than Busse et al.'s  *in vivo* estimate (600) |
| maximal IL-2 secretion rate | *IL2* | 300 min^−1^ | Busse et al. 2010 |
| maximal CD25 expression rate | *IL2R* | 100 min^−1^ | Busse et al. 2010 |
| IL2-IL2R association | *k_on_* | 600(µM min)^−1^ | Busse et al. 2010 |
| IL2-IL2R dissociation | *k*_off_ | 0.015 min^−1^  (t_1/2_ ≈ 45 min) | Smith 2006 |
| IL2-IL2R complex internalization | *k*_deg_ | 0.05 min^−1^  (t_1/2_ ≈ 15 min) | Smith 2006 |
